# Effects of low-intensity pulsed ultrasound and hyperbaric oxygen on human osteoarthritic chondrocytes

**DOI:** 10.1186/1749-799X-9-5

**Published:** 2014-02-05

**Authors:** Li-Jen Yuan, Chi-Chien Niu, Song-Shu Lin, Chuen-Yung Yang, Yi-Sheng Chan, Wen-Jer Chen, Steve WN Ueng

**Affiliations:** 1 Department of Orthopaedic Surgery, Chang Gung Memorial Hospital, 5, Fu-Hsin St. 333, Kweishan, Taoyuan 333, Taiwan; 2 Graduate Institute of Biomedical Sciences, Chang Gung University, 259 Wen-Hwa 1st Road, Taoyuan 333, Taiwan

**Keywords:** Low-intensity pulsed ultrasound, Hyperbaric oxygen, Osteoarthritis, TIMP-1, MMP-3, Aggrecan, Type-II collagen

## Abstract

**Background:**

Although the individual effects of hyperbaric oxygen (HBO) and low-intensity pulsed ultrasound (LIPUS) on osteoarthritic (OA) chondrocytes have been reported, the effects of HBO combined with LIPUS treatment are unknown.

**Methods:**

OA chondrocytes were obtained from patients undergoing knee replacement surgery. RNA was isolated for real-time polymerase chain reaction (PCR) analysis of inducible nitric oxide synthase (iNOS), type-II collagen, and aggrecan gene expression. The protein levels of MMP-3 and TIMP-1 were quantified by enzyme-linked immunosorbent assay (ELISA) after LIPUS or HBO treatment. The data are given as mean ± standard deviation (SD) of the results from three independent experiments. A *p* value less than 0.05 was defined as statistically significant.

**Results:**

Our data suggested that ultrasound and HBO treatment increased cell bioactivity of OA chondrocytes. Real-time PCR analysis showed that HBO treatment increased the mRNA of type-II collagen, aggrecan, and TIMP-1 but suppressed the iNOS expression of OA chondrocytes. LIPUS treatment increased the type-II collagen and iNOS expression of OA chondrocytes. ELISA data showed that HBO or LIPUS treatment increased TIMP-1 production of OA chondrocyte. MMP-3 production was suppressed by HBO treatment. HBO combined with LIPUS treatments resulted in additive effect in TIMP-1 production and compensatory effect in iNOS expression.

**Conclusion:**

HBO combined with LIPUS treatment-induced increase of the anabolic factor (TIMP-1)/catabolic factor (MMP-3) ratio may provide an additive therapeutic approach to slow the course of OA degeneration.

## Background

Mechanical stimulus is thought to be one of the important factors regulating chondrocyte metabolism [1]. Excessive mechanical stimulus has been reported to destroy articular cartilage directly and also induce other destructive factors [2]. Conversely, insufficient mechanical stimulus, such as that due to joint immobilization, has also been associated with cartilage destruction [3]. On the other hand, moderate (physiological) mechanical stimulus has been confirmed not only to promote articular cartilage anabolism [4] but also to inhibit catabolism [5,6].

Low-intensity pulsed ultrasound (LIPUS) is a representative therapy in the orthopedic field and is clinically used to treat fractures with nonunion and to promote bone union [7]. Application of high-intensity continuous ultrasound (1–300 W/cm^2^) generates considerable heat in living tissues. In contrast, LIPUS (<100 mW/cm^2^) has much lower intensities with nonthermogenic and nondestructive actions. Mechanical strains received in the skeleton result in the promotion of bone formation, possibly by inducing chondrocyte proliferation [8,9]. In this context, LIPUS has been shown to enhance the endochondral ossification in the healing process of fractured bones [10,11].

Ultrasound treatment has been tried as an approach to encourage cartilage repair [12]. Previous *in vitro* work has shown that the expression levels of integrins a5 and b1, as well as chondrocytic markers, Sox5, Sox9, collagen II, and aggrecan, were increased in chondrocytes exposed to a continuous ultrasound signal at 5.0 MHz (0.14 mW/cm^2^) [13]. Previous *in vivo* study in a New Zealand rabbit that modeled full-thickness osteochondral defects has demonstrated that exposure to LIPUS significantly improves the morphologic features and histologic characteristics of repaired cartilage [14]. Another *in vivo* experimental rat osteoarthritis model also demonstrated efficacy in cartilage restoration [15]. Exposure to LIPUS could significantly affect chondrocyte proliferation, phenotype expression, and matrix production; however, inconsistent effects were also observed.

Previous report suggested that VEGF induced by HBO is through c-Jun/AP-1 activation and through simultaneous activation of ERK and JNK pathways in umbilical vein endothelial cells [16]. HBO-suppressed ERK1/2 and p38 MAPK mediate nitric oxide-induced apoptosis on human degenerated intervertebral disc cells [17]. In OA chondrocytes, the MAP kinases, AP-1, and NF-κB transcription factors have been shown to play a predominant role in the expression of metalloproteinases (MMPs) and inflammatory genes and protein [18]. Our previous study demonstrated that attenuation of apoptosis and enhancement of proteoglycan synthesis in rabbit cartilage defects by HBO treatment are related to the suppression of IL-1β and nitric oxide (NO) production [19]. HBO treatment prevents NO-induced apoptosis in articular cartilage injury via enhancement of the expression of heat shock protein 70 [20].

Although the individual effect of HBO or LIPUS on the chondrocytes have been reported, the effect of HBO combined with LIPUS treatment is still controversial. We harvested the articular cartilage from patients who receive total knee arthroplasty (TKA). We investigate whether the beneficial effect on OA will be synergistic up-regulation (such as aggrecan, type-II collagen, and TIMP-1 expression) and the subversive effect will be complementary compensation (such as iNOs expression) after HBO combined with LIPUS treatment.

## Methods

The experimental protocol was approved by the Human Subjects Institutional Review Board of the Chang Gung Memorial Hospital.

### Cell isolation and cell culture

Articular cartilage specimens (tibial plateaus and femoral condyles) were obtained from 20 Ahlbäck grade IV or Kellgren and Lawrence grade IV OA patients who receive TKA surgery. The specimen was obtained under aseptic conditions, and the cartilage was dissected on ice. The chondrocytes were released from the articular cartilage by sequential digestion with 1 mg/ml collagenase (Sigma, St. Louis, MO, USA) in Dulbecco's minimal essential medium (DMEM/F-12) (Gibco, Grand Island, NY, USA) containing 5% fetal bovine serum (FBS) and incubated at 37°C until the fragments were digested. The isolated chondrocytes were centrifuged (1,000 rpm for 5 min), washed with PBS, and seeded in T-75 tissue culture flasks (Falcon, BD Biosciences, Drive Franklin Lakes, NJ, USA) in 15 ml of medium (DMEM/F-12) supplemented with 20% (*v*/*v*) FBS and antibiotics (mixture of 100 U/ml of penicillin and 100 μg/ml of streptomycin; Gibco). The cultures were incubated in a humidified atmosphere of 5% CO_2_/95% air until cell confluence.

### Cell exposure to intermittent HBO

About 3 × 10^4^ cells are platted on the 35-mm cell culture dish (Falcon) with medium (DMEM/F-12) containing 10% FBS and incubated at 37°C in a humidified atmosphere of 5% CO_2_ and 95% air. Control cells were maintained in 5% CO_2_/95% air (non-HBO) through the experimental protocol. All hyperoxic cells were exposed to 100% O_2_ for 25 min then to air for 5 min at 2.5 atmospheres absolute (ATA) in a hyperbaric chamber (Sigma II, Perry Baromedical Corporation, Riviera Beach, FL, USA) with a total treatment of 90 min per 48 h.

#### Cell exposure to LIPUS treatment

About 3 × 10^4^ cells are platted on the 35-mm cell culture dish with medium (DMEM/F-12) containing 10% FBS and incubated at 37°C in a humidified atmosphere of 5% CO_2_ and 95% air. A UV-sterilized transducer (Exogene 3000; Smith & Nephew Inc., Memphis, TN, USA) that generated 1.5-MHz US in a pulsed-wave mode (200-μs pulse burst width with repetitive frequency of 1 kHz at an intensity of 30 mW/cm^2^) was immersed vertically into each culture well and placed to just contact the surface of the medium. The distance between the transducer and the cells was approximately 5–6 mm. The exposure time was 20 min per 48 h.

#### Cell exposure to LIPUS combined with HBO

The cells were treated with LIPUS first and then with HBO as previously described.

#### RNA extraction and real-time PCR analysis

At 24 h after each treatment, cellular RNA was isolated using an RNeasy mini kit (Qiagen, Valencia, CA, USA) and reverse-transcribed into cDNA with the ImProm-II reverse transcription system (Promega, Madison, WI, USA). For real-time PCR detection of iNOs, aggrecan, and type-II collagen RNA transcripts, cDNA was analyzed on an ABI PRISM 7900 sequence detection system using the TaqMan PCR Master Mix (Applied Biosystems, Foster City, CA, USA). The cycle threshold (Ct) values were obtained, and the data was normalized to GAPDH expression using the ΔΔCt method to calculate relative mRNA levels of each target gene.

### MMP-3 ELISA assay

The cells were plated at 3 × 10^4^ cells per 35-mm tissue culture dish (Falcon) in 2.5 ml of medium (DMEM/F-12) containing 5% FBS. The level of TIMP-1 in the conditioned media after each treatment was determined using a commercial immunoassay kit (Quantikine Human TIMP-1, R&D System, Minneapolis, MN, USA). At intervals of 48, 96, and 144 h, 200 μl of conditioned media was accumulated and tested according to the manufacturer's instructions. The measurements were performed in triplicate.

### TIMP-1 ELISA assay

The cells were plated at 3 × 10^4^ cells per 35-mm tissue culture dish in 2.5 ml of medium (DMEM/F-12) containing 5% FBS. The level of TIMP-1 in the conditioned media after each treatment was determined using a commercial immunoassay kit (Quantikine Human TIMP-1, R&D System). At intervals of 48, 96, and 144 h, 200 μl of conditioned media was accumulated and tested according to the manufacturer's instructions. The measurements were performed in triplicate.

## Results

### Effect of HBO and LIPUS on MMP-3 production

**Figure 1:**
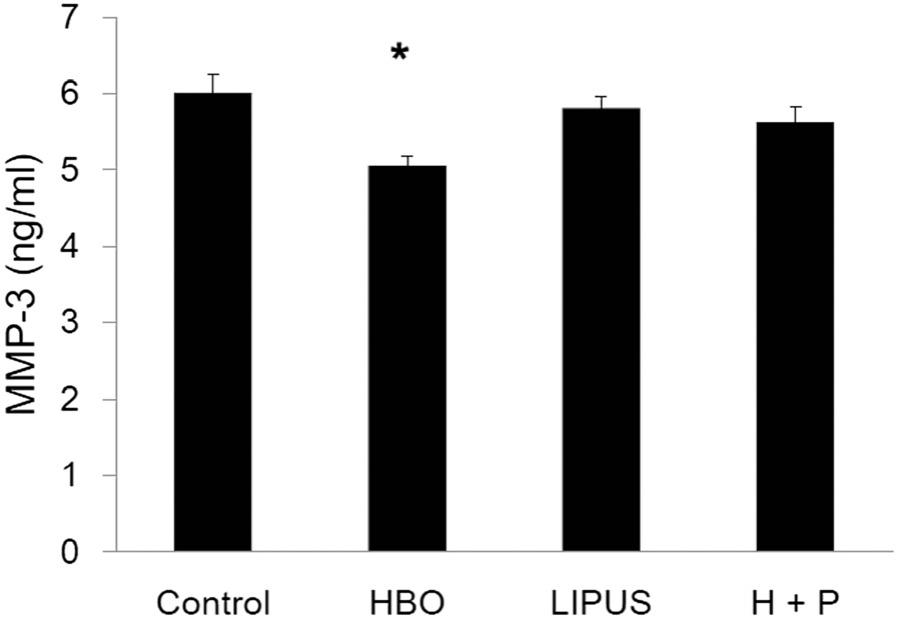
**Effect of HBO and LIPUS on MMP-3 production.** MMP-3 production in OA chondrocytes was significantly down-regulated by the HBO treatment (*p < 0.05).

Figure 1 shows the effect of HBO, LIPUS, and HBO combined with HBO on MMP-3 production (data are presented as mean ± SD; control group vs. HBO group: 6.01 ± 0.23 ng/ml vs. 5.05 ± 0.12 ng/ml, *p* < 0.05; control group vs. LIPUS group: 6.01 ± 0.23 ng/ml vs. 5.81 ± 0.15 ng/ml, *p* > 0.05; control group vs. ILPUS + HBO group: 6.01 ± 0.23 ng/ml vs. 5.62 ± 0.21 ng/ml, *p* > 0.05, *n* = 3). The MMP-3 production in OA chondrocytes was significantly down-regulated by the HBO treatment.

### Effect of HBO and LIPUS on TIMP-1 production

**Figure 2:**
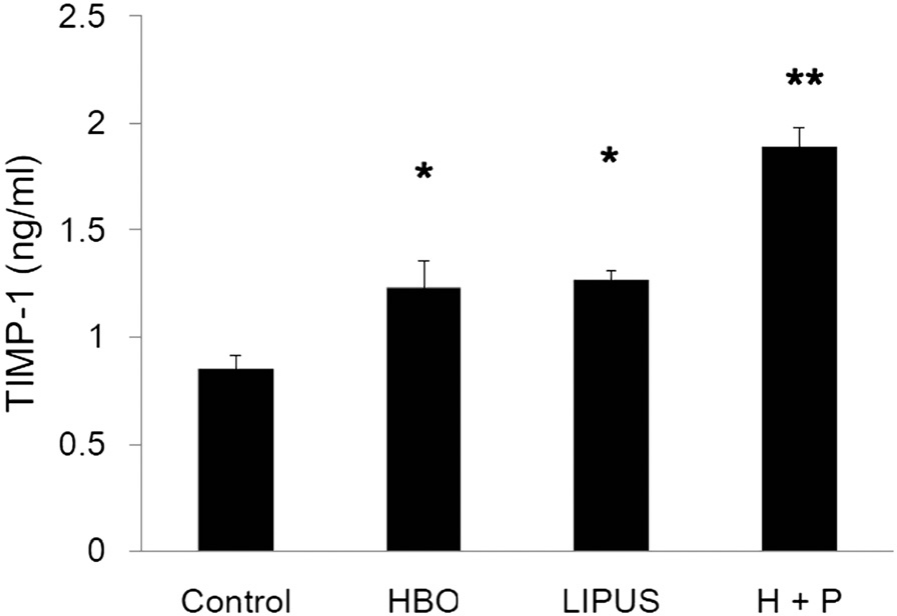
**Effect of HBO and LIPUS on TIMP-1 production.** TIMP-1 production in OA chondrocytes was significantly up-regulated by the HBO and LIPUS treatment. In addition, the HBO combined with LIPUS treatment resulted in an additive effect in the TIMP-1 production (*p < 0.05, **p < 0.01).

Figure 2 shows the effect of HBO, LIPUS, and HBO combined with HBO on TIMP-1 production (data are presented as mean ± SD; control group vs. HBO group: 0.85 ± 0.06 ng/ml vs. 1.23 ± 0.12 ng/ml, *p* < 0.05; control group vs. LIPUS group: 0.85 ± 0.06 ng/ml vs. 1.26 ± 0.05 ng/ml, *p* < 0.05; control group vs. LIPUS + HBO group: 0.85 ± 0.06 ng/ml vs. 1.89 ± 0.09 ng/ml, *p* < 0.01, *n* = 3). The TIMP-1 production in OA chondrocytes was significantly up-regulated by the HBO and LIPUS treatment. In addition, the HBO combined with LIPUS treatment resulted in an additive effect in the TIMP-1 production.

### Effect of HBO and LIPUS on gene expression of iNOs, aggrecan, and type-II collagen

**Figure 3:**
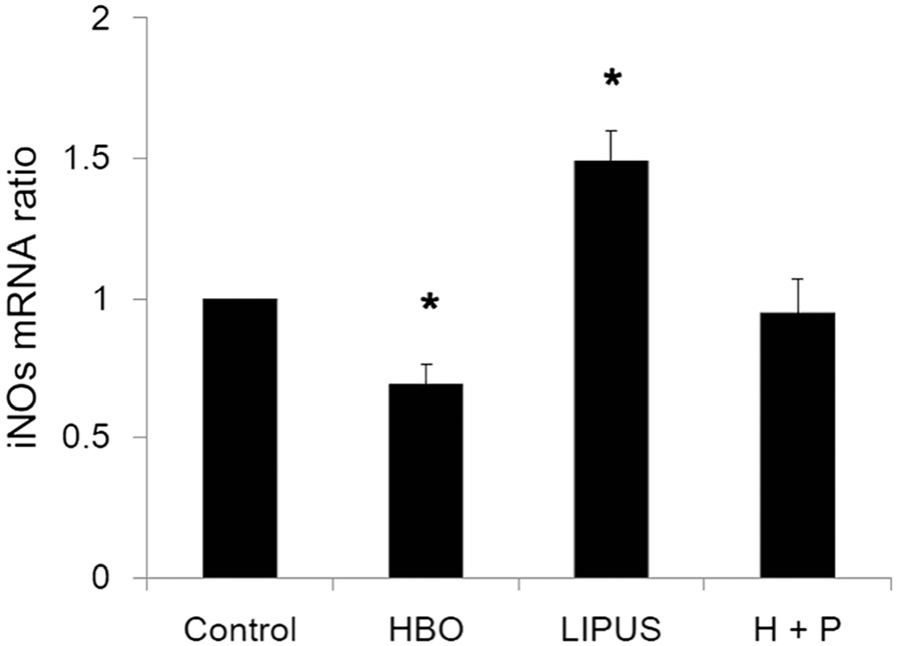
**Effect of HBO and LIPUS on transcription of iNOS.** HBO suppressed while LIPUS increased the gene expressions of iNOS in OA chondrocytes (*p < 0.05). HBO combined with LIPUS treatments resulted in compensatory effect in the iNOS expression.

**Figure 4:**
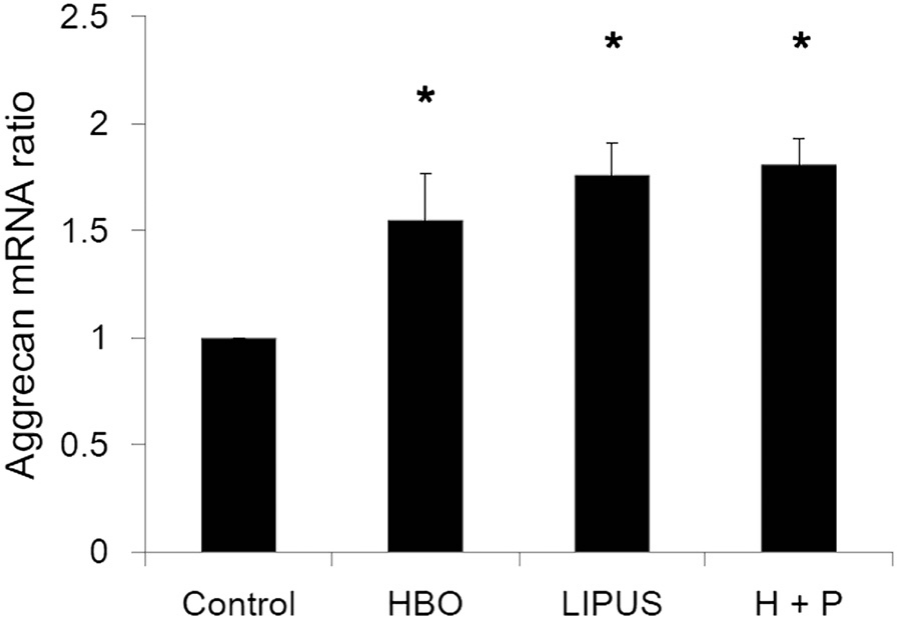
**Effect of HBO and LIPUS on transcription of aggrecan.** HBO or LIPUS treatment significantly increased the gene expressions of aggrecan (*p < 0.05) in OA chondrocytes as compared with the control cells.

**Figure 5:**
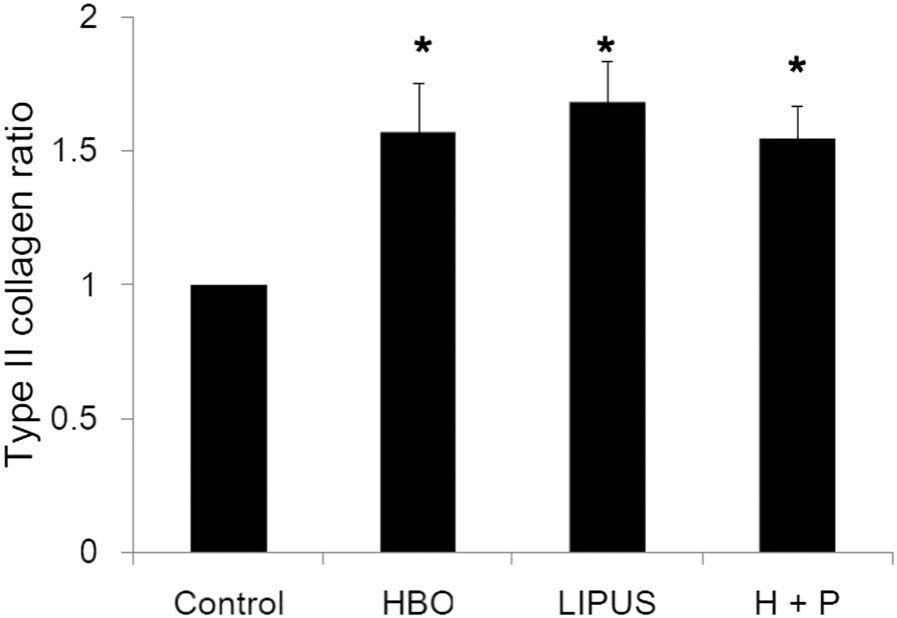
**Effect of HBO and LIPUS on transcription of type-II collagen.** HBO or LIPUS treatment significantly increased the gene expressions of type-II collagen (*p < 0.05) in OA chondrocytes as compared with the control cells.

Figures 3, 4, and 5 show the effects of HBO and LIPUS on transcription of iNOs (data are presented as mean ± SD; HBO group/control group: 0.69 ± 0.07 fold, *p* < 0.05; LIPUS group/control group: 1.49 ± 0.11 fold, *p* < 0.05; HBO + LIPUS group/control group: 0.95 ± 0.12 fold, *p* > 0.05; *n* = 3; Figure 3), aggrecan (data are presented as mean ± SD; HBO group/control group: 2.25 ± 0.32 fold, *p* < 0.05; LIPUS group/control group: 1.14 ± 0.15 fold, *p* > 0.05; HBO + LIPUS group/control group: 2.01 ± 0.12 fold, *p* < 0.05; *n* = 3; Figure 4), and type-II collagen (data are presented as mean ± SD; HBO group/control group: 1.57 ± 0.22 fold, *p* < 0.05; LIPUS group/control group: 1.68 ± 0.15 fold, *p* < 0.05; HBO + LIPUS group/control group: 1.51 ± 0.12 fold, *p* < 0.05; *n* = 3; Figure 5) in OA chondrocytes. HBO suppressed while LIPUS increased the gene expressions of iNOS in OA chondrocytes. HBO combined with LIPUS treatments resulted in compensatory effect in iNOS expression (Figure 3). HBO or LIPUS treatment significantly increased the gene expressions of aggrecan (Figure 4) and type-II collagen (Figure 5) in OA chondrocytes as compared with the control cells.

## Discussion

The secretion of proteolytic enzymes by the cartilage has been confirmed to contribute to the loss of extracellular matrix in OA. MMPs are capable of degrading the macromolecules of connective tissue matrices and have been considered the major proteases responsible for the pathologic destruction of tissue [21]. Moreover, an imbalance between MMPs and their inhibitors, tissue inhibitors of metalloproteinases (TIMPs), is responsible for the pathogenic sequence of cartilage degradation [22].

To elucidate whether mechanical stimulation by LIPUS combined with HBO treatment is chondrocyte-protective, we studied the effect of LIPUS combined with HBO treatment at several intensities on the protein expression of MMPs and TIMPs. LIPUS may potentially protect articular cartilage by inhibiting MMP-13 and MMP-1 mRNA expression in an intensity-dependent manner [23]. TIMP-1 mRNA expression was inhibited significantly by LIPUS stimulation of the articular cartilage explants at 67 mW/cm^2^ but up-regulated by the stimulation of the cultured chondrocytes at 30 mW/cm^2^[23]. In the present study, the MMP-3 production was significantly down-regulated by the HBO treatment but not by the LIPUS stimulation (Figure 1). The TIMP-1 production in OA chondrocytes was significantly up-regulated by the HBO or LIPUS treatment (Figure 2). We further showed that HBO combined with LIPUS treatment resulted in an additive effect in the TIMP-1 production (Figure 2). HBO combined with LIPUS treatment-induced increase of the anabolic factor (TIMP-1)/catabolic factor (MMP-3) ratio may provide a therapeutic approach to slow the course of OA chondrocyte degeneration.

Nitric oxide (NO) is a highly reactive nitrogen radical implicated in multiple biological processes, including regulation of vascular tone, platelet and leukocyte adhesion, neurotransmission, mediation of excessive vasodilatation, and cytotoxic actions of macrophages against microbes and tumor cells [24]. Ultrasound stimulates NF-κB activation and iNOS expression in cultured preosteoblasts [25]. Exposure to LIPUS increases NO and prostaglandin release, which are required for mechanically induced bone formation [26]. However, apoptosis of chondrocytes can be induced by NO [10,16,27]. HBO treatment prevents NO-induced apoptosis in articular cartilage injury via enhancement of the expression of heat shock protein 70 [20]. In the present study, HBO suppressed while LIPUS increased the gene expressions of iNOS in OA chondrocytes (Figure 3). HBO combined with LIPUS treatment resulted in compensatory effect in iNOS expression in OA chondrocytes (Figure 3), thus may prevent NO-induced apoptosis.

HBO treatment increased PG synthesis *in vivo*[19,20]. However, the effect of LIPUS to stimulate chondrocyte matrix synthesis is still controversial. Several *in vitro* studies have been undertaken to characterize the effects of LIPUS on chondrocytes in both monolayer and 3D model systems. These studies report the up-regulation of aggrecan and collagen II genes [28-30] and GAG synthesis [31]. However, conflicting reports suggest that LIPUS induces, at best, a transient effect on chondrocyte culture systems in terms of GAG and collagen II production [32] and aggrecan gene expression [33]. In the present study, similar results suggested that the aggrecan and type-II collagen mRNA expression in the OA chondrocytes were significantly up-regulated by HBO treatment. However, there was no additive effect in aggrecan and type-II collagen mRNA expression by HBO combined with LIPUS treatment (Figures 4 and 5).

In this paper, the author combined a chemical factor (hyperbaric oxygen) and a mechanical factor (LIPUS) treatment. The weighting of these two factors are equal in the combined treatment, and the induced increase of the (TIMP-1)/catabolic factor (MMP-3) ratio may provide an additive therapeutic approach to slow the course of OA degeneration. Although the effects of the combined factors are better than those of a single factor, the optimal combination ratio of these two factors needs further investigation.

## Conclusion

HBO combined with LIPUS treatment resulted in an additive effect in the TIMP-1 production and a compensatory effect in iNOS expression. Therefore, we will apply similar techniques of HBO combined with LIPUS therapy in future studies of cartilage injury models. The advantage of HBO combined with LIPUS treatment is that it is a useful tool for clinics and a more applicable clinical therapy.

## Competing interests

The authors declare that they have no competing interests.

## Authors' contributions

LJY, SWNU, and CCN designed the study. SSL and CYY analyzed and interpreted the data. CCN, YSC, and WJC provided the study materials and patients. SSL and SWNU drafted the manuscript. All authors read and approved the final manuscript.
